# Editorial: Novel technologies in the diagnosis and management of sleep-disordered breathing

**DOI:** 10.3389/frsle.2024.1385793

**Published:** 2024-03-07

**Authors:** Henri Korkalainen, Ding Zou

**Affiliations:** ^1^Department of Technical Physics, University of Eastern Finland, Kuopio, Finland; ^2^Diagnostic Imaging Center, Kuopio University Hospital, Kuopio, Finland; ^3^Center for Sleep and Vigilance Disorders, Institute of Medicine, Sahlgrenska Academy, University of Gothenburg, Gothenburg, Sweden

**Keywords:** sleep disorder, obstructive sleep apnea, machine learning, phenotyping, home sleep apnea testing, treatment, deep learning, precision medicine

The year 2023 is a memorable one in the history of sleep-disordered breathing (SDB). Obstructive sleep apnea (OSA) was first described as a new disease 50 years prior in 1973 (Guilleminault et al., 1973). After these 50 years of continuous development, nocturnal SDB has become one of the most active fields in sleep medicine, forming a platform for multidisciplinary collaboration.

## Sleep-disordered breathing diagnosis and management—Where are we now?

SDB encompasses a wide spectrum of conditions, from habitual snoring to severe cases of OSA, and affects a vast segment of the population worldwide (Benjafield et al., 2019). These conditions not only disrupt sleep but also lead to a range of negative health consequences such as excessive daytime sleepiness, cognitive impairments, and increased risk of cardiometabolic diseases. Traditional diagnostic methods, including in-lab polysomnography, often necessitate specialized sleep centers, are labor-intensive, and are not readily available. The current diagnostic methods also fall short in forecasting long-term health implications, underscoring an urgent need for innovation in diagnostic approaches and metrics for tailored disease management. Likewise, the management of SDB also stands at a transformative juncture in the rapidly advancing field of sleep medicine. While continuous positive airway pressure (CPAP) therapy remains the cornerstone of SDB treatment, its effectiveness in addressing the broader spectrum of SDB-related health issues is limited (Patil et al., 2024). This scenario underscores the critical need for alternative treatment modalities. The integration of machine learning, advanced diagnostic technologies, and novel therapeutic strategies heralds a move toward more personalized care and improved outcomes for patients (Korkalainen et al., 2024).

This Research Topic delves into the forefront of innovation in the diagnosis and management of SDB, highlighting a shift toward diagnostic tools and treatment methods that promise greater accuracy, efficiency, and patient-centric care. By exploring the latest in diagnostic technologies—ranging from portable devices capable of capturing detailed sleep data outside conventional lab settings to computational techniques designed to automate and enhance data analysis—this Research Topic underscores the move toward more accessible and comprehensive sleep assessments. In addition, the Research Topic examines cutting-edge approaches in the development of disease management, such as advanced physiological modeling and phenotyping, which aim to unravel the complexities of individual disease patterns, symptoms, and associated conditions. These insights are pivotal for developing personalized management strategies that address the nuanced needs of those affected by SDB. Together, the articles presented in this Research Topic offer a glimpse into the evolving landscape of SDB care, highlighting both the current hurdles and promising avenues for future interventions.

## At-home recordings for more affordable and available diagnostics

Accurate diagnosis is the cornerstone for assessing the severity of sleep disorders as well as for designing optimal disease management. New more affordable and simpler solutions are required due to the shortcomings of polysomnography. This can be made possible by technological advances as well as advanced analytical pathways, machine learning, and deep learning methodologies. Zou et al. discuss advancements in home sleep apnea testing that utilize technology based on peripheral arterial tone and photoplethysmography to detect respiratory events and their potential to act as a non-invasive, cost-effective, and accessible solution for multi-night sleep monitoring. Concurrently, Campbell and Sulaiman review the diagnostic potential of the WatchPAT device, which utilizes peripheral arterial tomography in conjunction with heart rate, oximetry, actigraphy, and respiratory movements for a minimally intrusive home diagnosis of OSA. This device exemplifies the trend toward integrating multiple physiological signals to refine diagnostic accuracy outside traditional laboratory environments. Teplitzky et al. contribute to this dialogue by reviewing alternative diagnostic methods to polysomnography that could increase the efficiency and accessibility of pediatric OSA diagnosis, highlighting the urgent need for adaptable diagnostic tools in younger populations. Finally, Zhu et al. introduce a novel approach using triboelectric nanogenerators embedded in wearable devices. This method has the potential for real-time, non-invasive sleep monitoring through analysis of respiratory rates and body movements.

## Leveraging machine learning to improve diagnostics

Artificial intelligence, driven by advancements in machine learning and deep learning methodologies further holds the potential to transform diagnostics. Overall, these approaches have shown promising results in automating the analysis of overnight recordings as seen in Somaskandhan et al.. This research shows the capability of a deep learning-based method to automatically score sleep stages in preadolescent populations, a population that often necessitates in-laboratory polysomnography. Their results already illustrate accuracy on par with inter-rater reliability between expert scorers. Meanwhile, Piccini et al. explore the application of machine learning to identify sleep stages and diagnose OSA using electrodermal activity signals. Their work suggests that wearable devices could significantly reduce reliance on polysomnography by providing a more patient-friendly approach to sleep diagnostics. In addition to automating the scoring or simplifying the recordings, deep learning and machine learning methods hold the potential to gain deeper insights from the recordings. This is supported by a review by Anderer et al. on the utilization of artificial intelligence to provide consistent and reliable scoring of sleep stages based on neurological and cardiorespiratory signals, especially the utilization of hypnodensity as a method to quantify sleep stage ambiguity and stability. These works offer a novel perspective on the assessment of SDB.

## Novel diagnostic metrics and measurement techniques

To unravel the complexities of sleep disorders, it is crucial to delve beyond traditional analyses by employing innovative methods that offer a more nuanced understanding of sleep dynamics and pathophysiological underpinnings. Traditional sleep stage classification, with its discrete five-stage system, while useful, often falls short in capturing the intricate patterns of sleep fragmentation seen in disorders like OSA. Recognizing this limitation, alternative analytical approaches have been developed to provide deeper insights into sleep quality and architecture. One of these methods is odds ratio product (ORP), a continuous metric for sleep depth that can help identify differences in sleep depth between and within individuals even when the changes are not discernible by conventional sleep staging. Younes, the developer of ORP, provides a comprehensive overview of ORP's measurement, validation, and application, underscoring its potential in identifying distinct phenotypes of sleep disorders. ORP thereby facilitates more personalized and effective management strategies for conditions such as SDB, insomnia, and idiopathic hypersomnia.

Innovative diagnostic tools such as mandibular jaw movements (MJM) and electrical impedance tomography can provide additional detailed and non-invasive information on sleep disorders. Martinot and Pépin review the use of MJM as a novel, non-invasive method for assessing respiratory effort in SDB. This approach utilizes a single point-of-contact sensor placed on the patient's chin to capture MJM, which is then analyzed using machine learning algorithms to provide diagnostic information. The article highlights the potential of MJM monitoring as a reliable, patient-friendly alternative to traditional, more invasive methods for measuring respiratory effort, such as esophageal pressure monitoring. Similarly, Piccin et al.'s exploration of neck electrical impedance tomography for monitoring upper airway patency during sleep offers real-time insights into airway obstruction dynamics, showcasing the potential of these methodologies in enhancing our diagnostic capabilities and ultimately improving patient care. Exploring the multifaceted relationship between sleep disorders and other physiological disturbances can further enrich our understanding. For instance, Ji et al.'s study on gastrointestinal electrophysiological signals reveals a promising avenue for predicting sleep disturbances, highlighting the interconnected nature of physiological systems and their impact on sleep health. Together, these advancements signal a shift toward a more comprehensive and nuanced approach to diagnosing and understanding sleep disorders, paving the way for innovative treatment modalities and improved patient outcomes.

## Pathophysiological factors, endotyping, and phenotyping to guide treatment

The intricate nature of SDB necessitates a nuanced understanding of its pathophysiology for the development of personalized treatment modalities. The comprehensive phenotyping and endotyping of OSA, leveraging a wealth of data from patient characteristics and diagnostic recordings, is pivotal to tailoring treatments to address specific disease traits, and can potentially lead to enhanced treatment adherence. McNicholas and Korkalainen discuss the complex pathophysiology and phenotypes of OSA as well as the translation to personalized treatment strategies. Novel diagnostic approaches are needed to gain a deeper insight into endotypical and phenotypical factors and to gauge the systemic effects of OSA. This likely requires adaptation to facilitate ambulatory and multi-night diagnostic studies, as well as simplification of recordings and the development of more detailed analyses. As a potential solution, Finnsson et al. provide an overview of the Endo-Phenotyping Using Polysomnography (PUP) method, a model-based tool for estimating endotypic traits from standard polysomnography. This method represents a significant step forward in the pursuit of precision medicine for OSA, offering a pathway to targeted treatments based on individual patient profiles. However, the authors also acknowledge the challenges that must be overcome to translate the PUP algorithm into clinical practice, indicating that further research and development are essential for realizing the full potential of the technology in the context of personalized medicine in OSA. Moreover, the upper airway muscles have no bony or cartilaginous support and are prone to collapse during sleep and the role of neuromuscular function in the pathogenesis and management of OSA is summarized by a group of experts led by Mehra et al.. In their consensus, a point-of-care model using novel electrodiagnostic technology for upper airway assessment is proposed. These discussions illuminate the path toward understanding the underlying pathophysiology of SDB and the importance of detailed phenotyping and endotyping in developing personalized treatment plans. Such an approach not only promises to address the specific characteristics of the disease but also to improve patient outcomes through higher adherence to tailored treatment strategies.

The analysis of pathophysiological factors, endotypes, and phenotypes could guide the optimal treatment, as discussed extensively in Gruenberg et al. who highlight the potential benefits and limitations of various non-CPAP therapeutic modalities including myofunctional therapy, upper airway training, and several forms of electrical stimulation of the upper airway muscles and nerves. For SDB patients exhibiting high loop gain, CPAP may not suffice, prompting the need for alternative treatments such as the enhanced expiratory rebreathing space (EERS), reviewed by Quinn et al. EERS modifies the dead space of CPAP devices to mitigate CO_2_ loss during sleep arousals, thus stabilizing breathing patterns and addressing central sleep events triggered by hypocapnia. This approach provides an alternative for patients who are unresponsive to traditional CPAP therapy. Finally, Gentina et al. present the study protocol and baseline data for a prospective study (Self-Efficacy Measure for Sleep Apnea study; SEMSAS) underscoring the importance of health literacy, self-efficacy, and socio-economic factors in predicting CPAP adherence. This ongoing study promises to elucidate the multifaceted influences on CPAP compliance, offering a foundation for identifying patients most likely to benefit from CPAP and refining treatment approaches in the long term.

## Treatment innovations

Patients with central sleep apnea (CSA) are often multi-morbid and difficult to treat. Javaheri et al. evaluate the efficacy of phrenic nerve stimulation as a treatment alternative. Their findings suggest phrenic nerve stimulation is a safe and effective treatment modality, capable of reducing the apnea-hypopnea index (AHI) and enhancing sleep quality, marking it as a viable option for CSA management. Pediatric populations, particularly those with Down syndrome, also present unique challenges in OSA management. Liu et al.'s systematic review and meta-analysis of hypoglossal nerve stimulation highlights its potential selected patients, addressing the low CPAP adherence rates and the need for additional interventions post-surgery in this demographic. Additionally, advancements in mandibular advancement splint (MAS) therapy, as reviewed by Mohammadieh et al., showcase a new generation of MAS devices that integrate digital technologies and machine learning to improve treatment efficacy, patient selection, and compliance monitoring. Emphasizing the role of customization and technological integration in enhancing therapeutic outcomes, it is anticipated that MAS therapy will play a more important role in OSA management.

There is no pharmacological treatment for OSA in clinical practice. The combination of noradrenergic and antimuscarinic drugs discussed by Taranto-Montemurro et al. may open new avenues for management strategies. Despite the promise of these treatments, challenges remain in assessing disease severity and pinpointing specific treatment targets, underscoring the need for continued research and development in pharmacotherapy for OSA (Hedner and Zou, 2022). Together, these recent developments represent a shift toward personalized and diversified treatment strategies for OSA, addressing the limitations of CPAP therapy and expanding the therapeutic landscape to accommodate patient-specific needs and preferences.

## Conclusion

This is an exciting era in sleep medicine and sleep research with new concepts and innovations. As we stand on the precipice of transformative advancements in the diagnosis and management of SDB, the horizon is both promising and fraught with challenges. The integration of machine learning, advanced diagnostic modalities, and innovative treatment approaches heralds a new chapter of personalized medicine, tailored to meet the unique needs of individuals. The shift from “AHI medicine” and “one size fits all” concepts toward precision medicine is poised to revolutionize patient care, offering more accurate diagnoses, enhanced treatment efficacy, and improved patient adherence (Arnardottir et al., 2022). The successful translation of these advancements from research to clinical practice requires not only further validation through large-scale studies but also a reevaluation of existing healthcare models to ensure accessibility. Moreover, the adoption of new technologies necessitates comprehensive training for healthcare professionals to maximize the benefits of these tools. Interdisciplinary collaboration will be crucial in overcoming these barriers, uniting experts from fields such as sleep medicine, respirology, neurology, cardiology, otolaryngology, odontology, biomedical engineering, and data science to address the complex challenges ahead. As we navigate these uncharted waters, the collective efforts of the scientific and medical communities will be paramount in realizing the full potential of these innovations, ultimately improving the lives of millions suffering from SDB.

## Author contributions

HK: Conceptualization, Writing—original draft. DZ: Conceptualization, Writing—review & editing.
